# Announcement for 1890

**Published:** 1889-12

**Authors:** 


### HOMŒOPATHIC PHYSICIAN,

A MONTHLY JOURNAL OF

HOMŒOPATHIC MATERIA MEDICA AND CLINICAL MEDICINE.

If our school ever give up the strict inductive method of Hahnemann, we are lost, and deserve only to be mentioned as a caricature in the history of medicine."—CONSTANTINE HERING.

Vol. IX. DECEMBER, 1889. No. 12.

### ANNOUNCEMENT FOR 1890.

THE HOMŒOPATHIC PHYSICIAN will hereafter be conducted by Drs. Walter M. James and George H. Clark, Dr. Edmund J. Lee, so long its editor, retiring from the management. A corps of contributing editors, whose names will appear upon the title-page, will assist in maintaining the character of the journal. This enlargement of the editorial corps is done for the purpose of enlisting the more active aid of the physicians named. It will be the purpose of the new management to enlarge and expand their field of usefulness by being represented in each important centre of Homœopathy by an able and true Hahnemannian. THE HOMŒOPATHIC PHYSICIAN has long been known as the only exponent of Hahnemannian Homœopathy and as the one journal whose efforts have been solely directed to teaching the principles and the practice of medicine as developed by Samuel Hahnemann.

It will be the purpose of the new management to make the journal a teacher of homœopathic materia medica and clinical medicine; to this end they hope to devote their pages rather than to controversial essays. In their aid we bespeak the help of all our subscribers and contributors. The profession must ever bear in mind that editors do not and cannot make a journal; each subscriber must consider himself as under direct obligation to contribute of his learning and experience to the general supply of knowledge. THE HOMŒOPATHIC PHYSICIAN will be hereafter published chiefly as a journal of materia medica; it will be a forty-eight paged monthly journal; the subscription price will continue to be only two dollars and fifty cents ($2.50), *payable in advance.* Subscribers, contributors, exchanges, etc., will please send all their communications, as heretofore, directed to THE HOMŒOPATHIC PHYSICIAN, No. 1125 Spruce Street, Philadelphia.

THE HOMŒOPATHIC PHYSICIAN began its career in January, 1881. The sole object and purpose of its work was to show (as was then stated) that "the conscientious practitioner preserves intact the 'strict inductive method of Hahnemann,' also that the following are the true and essential features of Homœopathy:

The Law of the Similars,
The Single Remedy,
The Minimum Dose, the first being the unfailing law, the last two its logical corollaries." How clearly these ends have been kept in view, and how forcibly advocated, we leave our readers to judge. It may be truthfully said that the editors of THE HOMŒOPATHIC PHYSICIAN have always striven to uphold and to teach the philosophy of true Hahnemannian Homœopathy, and have never knowingly or willfully published any article which was non-homœopathic in its tone and teaching.

The editors wish to tender to their many friends and contributors their grateful thanks for all kindnesses received from them. No journal ever had warmer or more helpful friends. It is to be hoped these same friends will continue their support to the new management.

